# Thyroid function and ischemic heart disease: a Mendelian randomization study

**DOI:** 10.1038/s41598-017-07592-z

**Published:** 2017-08-17

**Authors:** Jie V. Zhao, C. Mary Schooling

**Affiliations:** 1 School of Public Health, Li Ka Shing Faculty of Medicine, The University of Hong Kong, Hong Kong, SAR China; 2 City University of New York, School of Public Health and Health Policy, New York, NY USA

## Abstract

To clarify the role of thyroid function in ischemic heart disease (IHD) we assessed IHD risk and risk factors according to genetically predicted thyroid stimulating hormone (TSH), free thyroxine (FT4) and thyroid peroxidase antibody (TPOAb) positivity. Separate-sample instrumental variable analysis with genetic instruments (Mendelian randomization) was used in an extensively genotyped case (n = 64,374)-control (n = 130,681) study, CARDIoGRAMplusC4D. Associations with lipids, diabetes and adiposity were assessed using the Global Lipids Genetics Consortium Results (n = 196,475), the DIAbetes Genetics Replication And Meta-analysis case (n = 34,380)-control (n = 114,981) study, and the Genetic Investigation of ANthropometric Traits (body mass index in 152,893 men and 171,977 women, waist-hip ratio in 93,480 men and 116,741 women). Genetically predicted thyroid function was not associated with IHD (odds ratio (OR) per standard deviation for TSH 1.05, 95% confidence interval (CI) 0.97 to 1.12; for FT4 1.01, 95% CI 0.91 to 1.12; for TPOAb positivity 1.10, 95% CI 0.83 to 1.46) or after Bonferroni correction with risk factors, except for an inverse association of FT4 with low-density lipoprotein-cholesterol. The associations were generally robust to sensitivity analyses using a weighted median method and MR Egger. This novel study provides little indication that TSH, FT4 or TPOAb positivity affects IHD, despite potential effects on its risk factors.

## Introduction

Cardiovascular disease (CVD) is the leading cause of mortality globally^1^. Despite substantial progress in prevention and control, the etiology of CVD is not completely understood^2^, as evidenced by failures of new treatments^3^. Moreover, men have substantially higher rates of ischemic heart disease (IHD) than women at the same level of established risk factors^2^, generating the possibility of discovering new potentially modifiable risk factors, possibly related to sex or gender, for a leading cause of death. Evolutionary biology suggests growth and reproduction trade-off against longevity^4^. Increasing genetic evidence in humans is emerging, indicating that lower growth hormone and/or insulin like growth factor (IGF) are associated with longer life and lower risk of major chronic diseases^5, 6^. Across the animal kingdom it is well-established that suppressing the reproductive axis increases lifespan, and promoting the reproductive axis reduces lifespan^7, 8^. However, the relation of the reproductive axis to lifespan has been little investigated in humans^9^. Estrogen administration has been shown to have no benefit for CVD in men^10^ or women^11^, while the role of testosterone remains controversial^12^, although both Health Canada and the Food and Drug Administration in the US have warned of the cardiovascular risk of testosterone in men^13, 14^, and testosterone increases the hallmark of atherosclerosis, i.e., coronary artery plaque volume^15^. The hypothalamic–pituitary–thyroid axis interacts with the hypothalamic–pituitary–gonadal axis^16^. Thyroid hormones are also involved in testicular development in animals^17^. In humans, both overt and subclinical thyroid dysfunction, especially the former, are associated with higher risk of CVD events^18–20^. As such, thyroid function might play a role in CVD.

Clinically, thyroid function is assessed by measuring serum thyroid stimulating hormone (TSH) and free thyroxine (FT4). TSH is a key regulator of thyroid function, which promotes the synthesis and secretion of thyroid hormones, i.e., thyroxine and triiodothyronine^21^. FT4 is the active form of thyroxine. Thyroid peroxidase antibodies (TPOAbs) also play a key role in the synthesis of thyroid hormones; TPOAb positivity is associated with higher risk of autoimmune hyperthyroidism^22^. Observationally, higher TSH, even within the normal range, is associated with higher risk of CVD events^23^. In some, but not all observational studies, higher TSH and lower FT4 are associated with unhealthier lipids and glucose metabolism, such as an association of higher TSH with higher total cholesterol^24^, higher low-density lipoprotein (LDL)-cholesterol^24^, lower high-density lipoprotein (HDL)-cholesterol^25^, and higher HbA_1c_^25^, and of higher FT4 with lower LDL-cholesterol^26^, higher HDL-cholesterol^27^, and lower fasting glucose^26^. However, some studies have found no association^27–31^. Observationally, people who are TPOAb-positive have higher TSH and faster carotid intima media thickness (cIMT) progression^32^, however, in another study, cardiovascular risk associated with subclinical hypothyroidism did not differ by TPOAb status^33^. A meta-analysis of cohort studies has shown that higher and lower TSH are both associated with higher risk of CVD events^34^. In several randomized controlled trials (RCTs), levothyroxine, the artificial thyroxine which lowers TSH via negative feedback loop, exerts beneficial effects on CVD risk factors^35–39^. For example, lowering TSH into the normal range with levothyroxine decreases brachial-ankle pulse wave velocity^35^, total cholesterol^36–38^, LDL-cholesterol^36–38^, apolipoprotein B^38^, and cIMT^37^. However, in the Coronary Drug Project, dextrothyroxine, the D-type isomer of levothyroxine, which is also a TSH-lowering agent, reduced cholesterol but increased the risk of CVD, so that the trial was discontinued because of excess risk^40^. However, dextrothyroxine and levothyroxine have quite different biological activity^41, 42^. As such, the role of thyroid function as a target of intervention in CVD is unclear. Two RCTs to examine the effect of levothyroxine on CVD are being proposed^43, 44^, which ideally should be preceded by genetic validation of role of thyroid function in CVD.

Mendelian randomization (MR) provides a means of assessing genetic validity, by examining whether people with genetically different levels of TSH, FT4 and TPOAb positivity have higher or lower risk of IHD. MR uses genetic endowment randomly allocated at conception as instrument to predict exposures, analogous to the randomization in RCTs, as such MR is an increasingly popular means of obtaining unconfounded estimates^45^. Thyroid function has high heritability (up to 65% for TSH and FT4 in twin and family studies^46^), which also provides a good opportunity to apply MR. To clarify the role of thyroid function, we assessed the associations of genetically predicted TSH, FT4 and TPOAb positivity with coronary artery disease/myocardial infarction (CAD/MI) using instrumental variable analysis with genetic instruments, i.e., MR, in large case-control studies with extensive genotyping. We similarly assessed the associations with established CVD risk factors, including lipids, diabetes and adiposity to identify if any associations with IHD were independent of these risk factors.

## Results

We identified 34 single nucleotide polymorphisms (SNPs) in the genes *PDE8B*, *PDE10A*, *CAPZB*, *MAF*, *VEGFA*, *NR3C2*, *IGFBP5*, *SOX9*, *NFIA*, *FGF7*, *PRDM11*, *MIR1179*, *INSR*, *ABO*, *ITPK1*, *NRG1*, *MBIP*, *SASH1*, *GLIS3*, *IGFBP2*, *SYN2*, *FOXE1*, and *GNAS* for TSH^47–49^, 7 SNPs in the genes *DIO1*, *LHX3*, *FOXE1*, *AADAT* and *B4GALT6* for FT4 in men and women^47, 49^, and 3 SNPs in the genes *TPO*, *MAGI3* and *KALRN* for TPOAb positivity^22^. Eight SNPs (rs12410532, rs28435578, rs2046045, rs6923866, rs2396084, rs3008034, rs116552240, and rs17767742) for TSH were excluded because of linkage disequilibrium (LD) with other SNPs for TSH, i.e., rs10799824, rs10032216, rs6885099, rs11755845, rs9472138, rs753760, rs657152, and rs3813582. Three SNPs (rs13015993 in *IGFBP5*, rs7568039 in *IGFBP2* and rs657152 in *ABO*) were excluded from the analysis of TSH with CAD/MI due to potential pleiotropy. Rs13015993 in *IGFBP5* and rs7568039 in *IGFBP2* might be associated with CAD/MI other than via TSH^50^ because associations with CAD were suggested by the MR Catalogue (p value 1.73 × 10^−4^ for rs13015993; 1.40 × 10^−4^ for rs7568039). Moreover, the expression of *IGFBP2* and *IGFBP5* relates to the growth hormone/IGF system^47^, which is associated with risk of major chronic diseases in genetic studies^5, 6^. Correspondingly, calorie restriction in a clinical trial also lowered IGF-1 and CVD risk factors^51^. Rs657152 is in *ABO*, a gene strongly associated with IHD for reasons that are not entirely clear but may be unrelated to TSH^52^. Three SNPs were not in CARDIoGRAMplusC4D Metabochip and 1 SNP was not in CARDIoGRAMplusC4D 1000 Genomes, and no proxies could be found (Supplemental Table 1). As such, 20 SNPs (mean F statistic 59.3) were used for TSH and CAD/MI in CARDIoGRAMplusC4D Metabochip and 22 SNPs (mean F statistic 58.3) in CARDIoGRAMplusC4D 1000 Genomes. Two FT4-related SNPs (rs11103377 and rs7694879) were excluded due to LD with rs7860634 and rs11726248 respectively. None were excluded due to pleiotropic associations with CAD/MI, so 5 SNPs (mean F statistic 61.4) were used for FT4. One TPOAb positivity SNP (rs653178) was excluded due to pleiotropic associations with blood pressure and CAD/MI^50, 53^, so 2 SNPs (mean F statistic 33.4) were used for TPOAb positivity and CAD/MI. Supplemental Table 1 gives the genetic associations with TSH, FT4 and TPOAb positivity, and the information for potential pleiotropy. Sex-specific SNPs were used in sex-specific analysis of adiposity in GIANT. For TSH, 6 SNPs reached genome-wide significance in men and 7 SNPs reached genome-wide significance in women. Three SNPs (rs13015993, rs753760, and rs10519227) in men and 2 SNPs (rs753760 and rs9497965) in women were excluded due to pleiotropic associations with obesity (Supplemental Table 1), as such, 3 SNPs in men and 5 SNPs in women were used for TSH and adiposity (Supplemental Table 2). For FT4, 3 SNPs reached genome-wide significance in men and 3 SNPs reached genome-wide significance in women. None were excluded due to LD or pleiotropy (Supplemental Table 2).

**Table 1:** Associations per standard deviation (SD) thyroid-stimulating hormone (TSH), free thyroxine (FT4) and thyroid peroxidase antibody (TPOAb) positivity with IHD and MI, obtained from separate sample instrumental variable analysis in CARDIoGRAMplusC4D Metabochip and CARDIoGRAMplusC4D 1000 Genomes, using different SNP selection and methods.

Exposure	outcome	CARDIoGRAMplusC4D Study	No. of SNPs	Methods	SNPs without pleiotropy^*^				No. of SNPs	All SNPs^‡^		Cochran’s Q (p value)	Intercept p value^†^
					OR	95% CI	Cochran’s Q (p value)	Intercept p value^†^		OR	95% CI		
TSH	IHD	Metabochip	20	IVW	1.05	0.97 to 1.12	21.7		22	1.07	0.997 to 1.14	28.5 (0.13)	
				WM	1.05	0.94 to 1.16	(0.30)			1.06	0.95 to 1.18		
				MR Egger	1.02	0.94 to 1.10		0.05		1.07	0.98 to 1.16		0.63
	IHD	1000	22	IVW	0.99	0.93 to 1.05	16.2		25	0.96	0.91 to 1.01	51.2	
		Genomes		WM	1.004	0.93 to 1.09	(0.76)			0.95	0.88 to 1.03	(0.001)	
				MR Egger	0.99	0.93 to 1.05		0.93		0.97	0.88 to 1.06		0.68
	MI	1000	22	IVW	1.005	0.94 to 1.07	14.5 (0.85)		25	0.98	0.92 to 1.04	53.4	
		Genomes		WM	0.99	0.90 to 1.08				0.98	0.90 to 1.07	(0.001)	
				MR Egger	1.001	0.94 to 1.07		0.78		1.001	0.89 to 1.12		0.50
FT4	IHD	Metabochip	5	IVW	1.01	0.91 to 1.12	3.6 (0.47)		5	1.01	0.91 to 1.12	3.6 (0.47)	
				WM	1.02	0.89 to 1.17				1.02	0.89 to 1.17		
				MR Egger	1.04	0.71 to 1.50		0.88		1.04	0.71 to 1.50		0.88
	IHD	1000	5	IVW	1.06	0.97 to 1.15	3.8 (0.44)		5	1.06	0.97 to 1.15	3.8 (0.44)	
		Genomes		WM	1.07	0.96 to 1.20				1.07	0.96 to 1.20		
				MR Egger	1.36	0.87 to 2.13		0.26		1.36	0.87 to 2.13		0.26
	MI	1000	5	IVW	1.06	0.96 to 1.17	3.9 (0.41)		5	1.06	0.96 to 1.17	3.9 (0.41)	
		Genomes		WM	1.07	0.95 to 1.21				1.07	0.95 to 1.21		
				MR Egger	1.62	0.98 to 2.67		0.09		1.62	0.98 to 2.67		0.09
TPOAb positivity	IHD	Metabochip	2	IVW	1.10	0.83 to 1.46	2.4 (0.12)		3	**1.41**	**1.10 to 1.80**	12.8 (0.002)	
				WM	1.11	0.82 to 1.51				**1.61**	**1.02 to 2.56**		
	IHD	1000 Genomes	2	IVW	0.95	0.79 to 1.13	0.05 (0.83)		3	1.07	0.91 to 1.26	18.4 (0.0001)	
				WM	0.95	0.78 to 1.14				0.95	0.79 to 1.16		
	MI	1000 Genomes	2	IVW	0.93	0.77 to 1.13	0.5 (0.49)		3	1.07	0.89 to 1.28	20.3 (<0.0001)	
				WM	0.93	0.75 to 1.15				0.96	0.77 to 1.19		

OR, odds ratio; MI, myocardial infarction; CI, confidence interval; IVW, inverse variance weighting; TPOAb, thyroid peroxidase antibody; WM, weighted median method.

^*^SNPs with potential pleiotropy for TSH: rs13015993, rs7568039 and rs657152; for TPOAb positivity: rs653178. No SNPs were identified to be with potential pleiotropy for FT4.

^†^The intercept can be interpreted as an estimate of the average pleiotropic effect across the genetic variants where a corresponding p-value of <0.05 indicates the presence of directional pleiotropy across the genetic variants included in the analyses.

^‡^ For TSH^,^ rs12523579 was used as a proxy for rs2928167. For FT4, rs4799592 was used as a proxy for rs113107469 in CARDIoGRAMplusC4D Metabochip; rs4297160 was used as a proxy for rs7045138 in CARDIoGRAMplusC4D 1000 Genomes.

**Figure 1:**
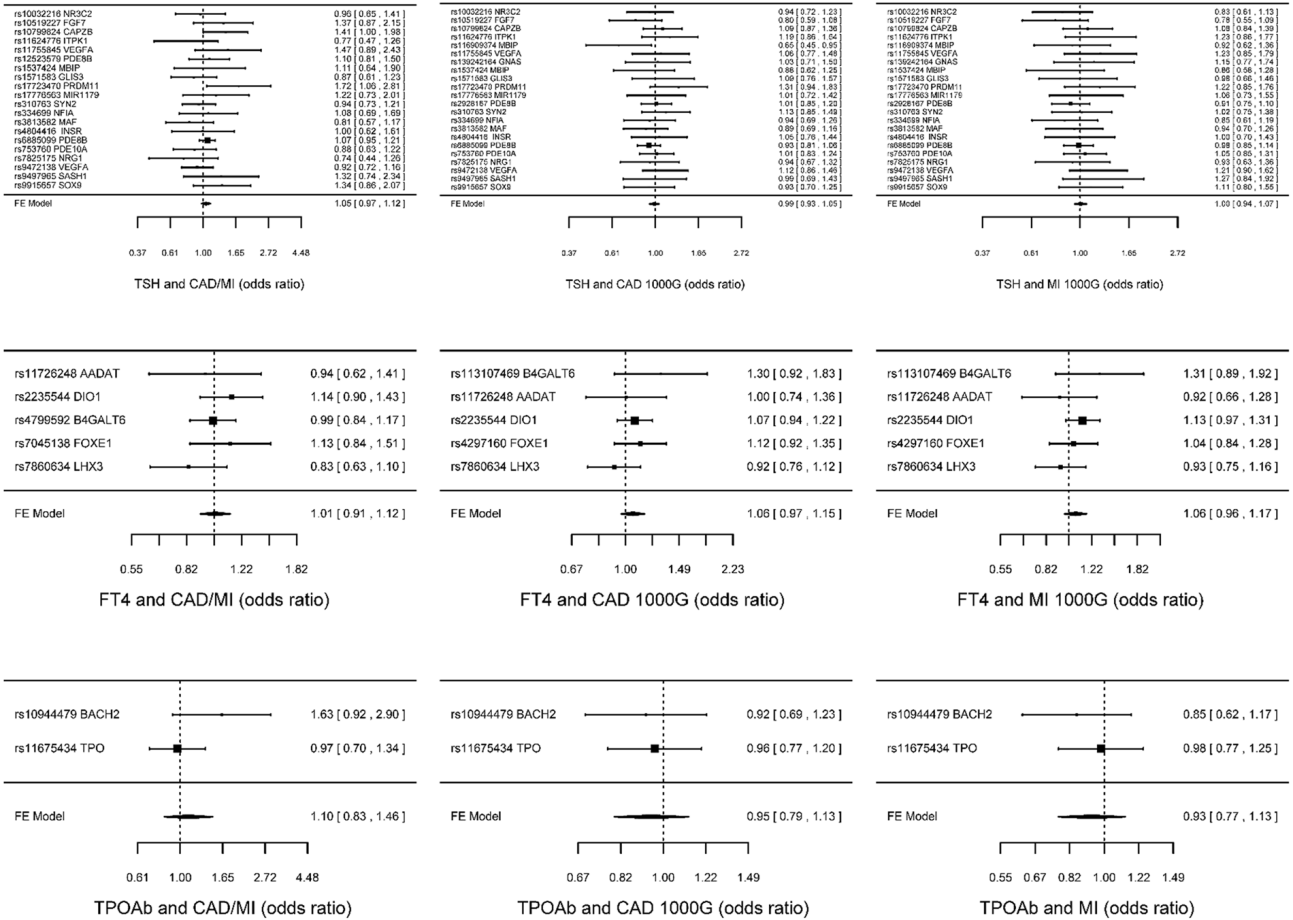
SNP specific and total associations per standard deviation (SD) thyroid-stimulating hormone (TSH), free thyroxine (FT4) and thyroid peroxidase antibody (TPOAb) positivity with CAD/MI, CAD and MI, obtained from separate sample instrumental variable analysis in CARDIoGRAMplusC4D^52, 58^ and CARDIoGRAMplusC4D 1000 Genomes^50^.

**Figure 2:**
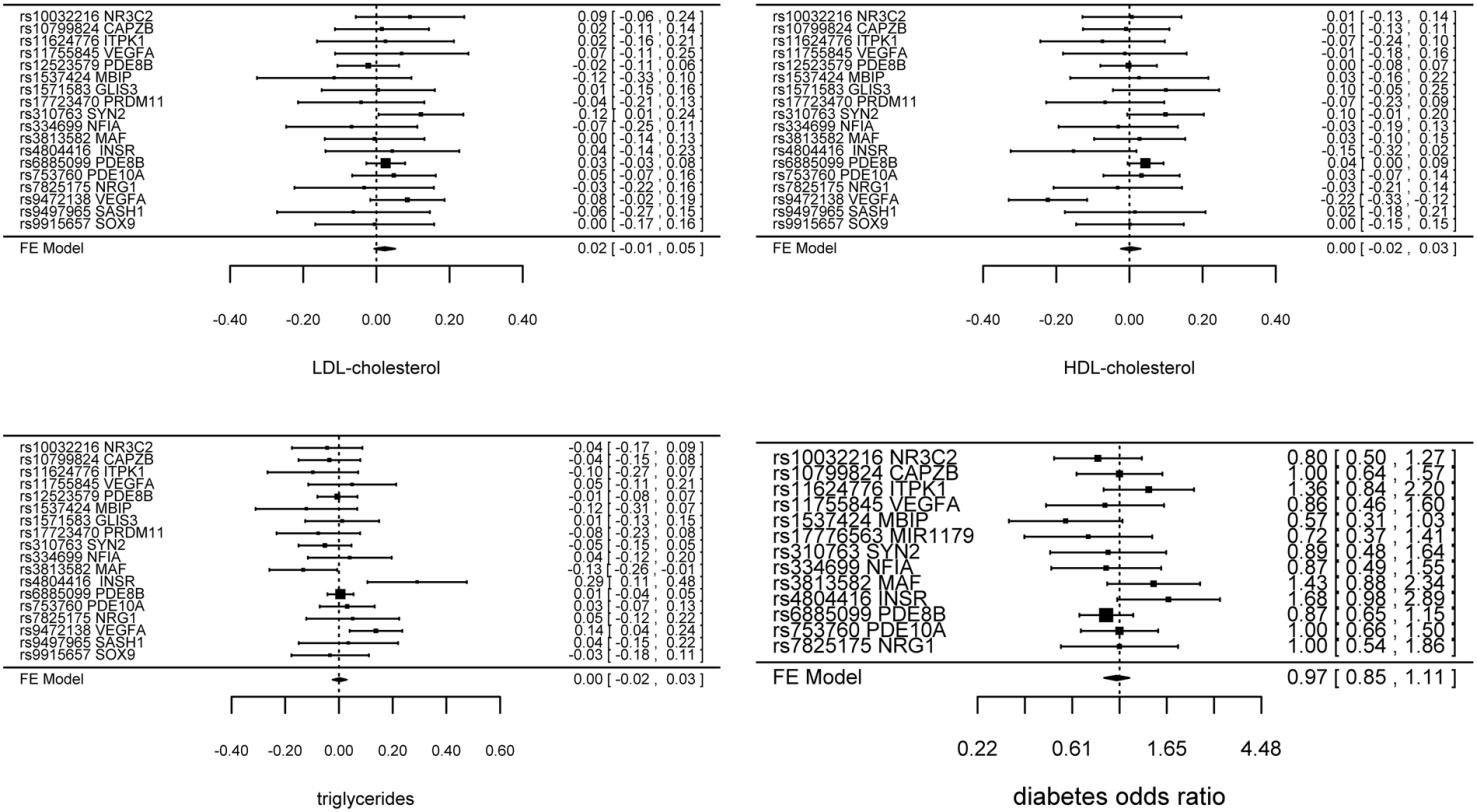
SNP specific and total associations per standard deviation (SD) thyroid-stimulating hormone (TSH) with low-density lipoprotein (LDL)-cholesterol (inverse normal transformed effect size), high-density lipoprotein (HDL)-cholesterol (inverse normal transformed effect size), triglycerides (inverse normal transformed effect size) and diabetes, obtained from separate sample instrumental variable analysis in the Global Lipids Genetics Consortium Results^59^ and DIAGRAM^60^.

**Figure 3:**
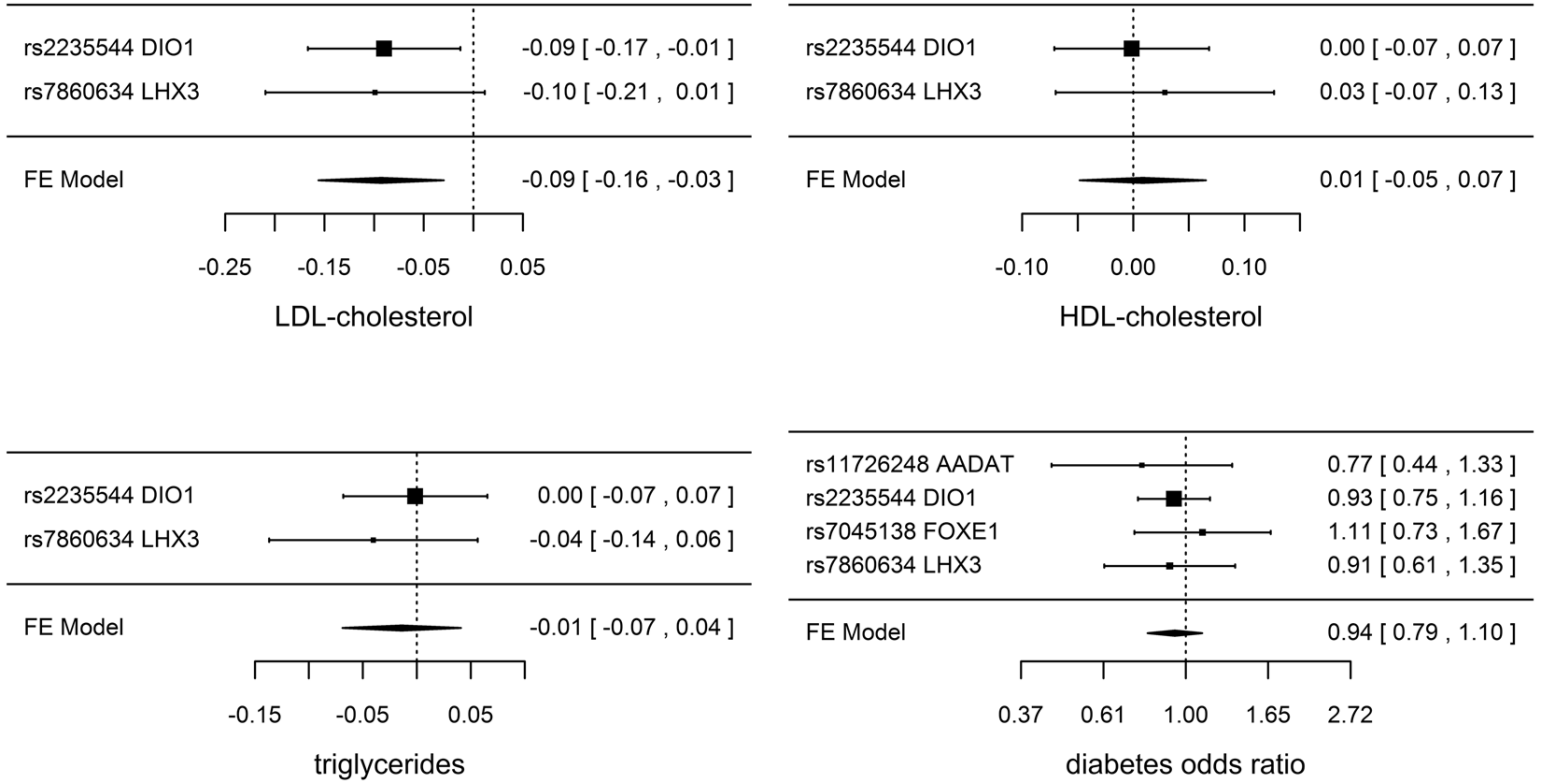
SNP specific and total associations per standard deviation (SD) free thyroxine (FT4) with low-density lipoprotein (LDL)-cholesterol (inverse normal transformed effect size), high-density lipoprotein (HDL)-cholesterol (inverse normal transformed effect size), triglycerides (inverse normal transformed effect size) and diabetes, obtained from separate sample instrumental variable analysis in the Global Lipids Genetics Consortium Results^59^ and DIAGRAM^60^.

**Figure 4:**
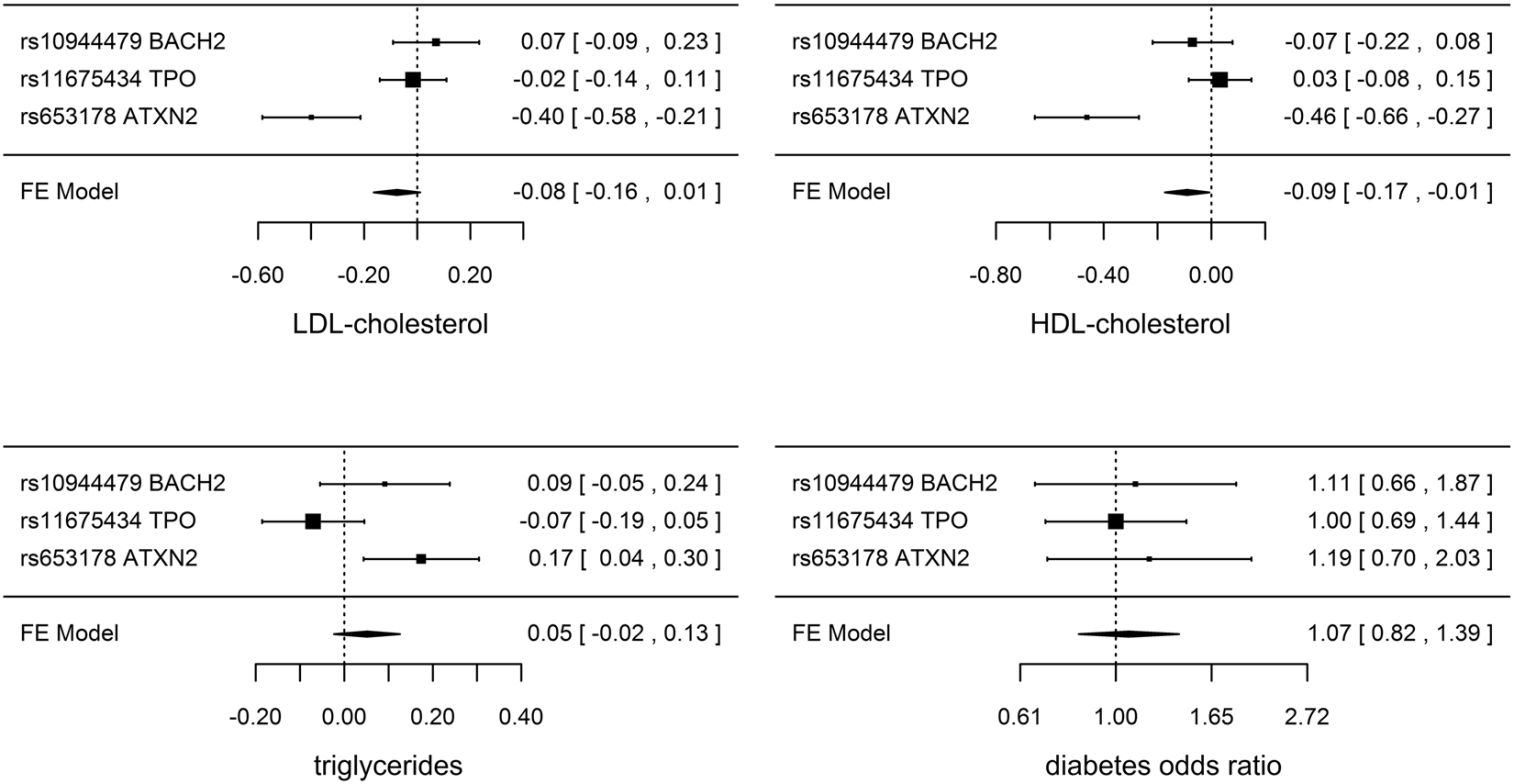
SNP specific and total associations of thyroid peroxidase antibody (TPOAb) positivity with low-density lipoprotein (LDL)-cholesterol (inverse normal transformed effect size), high-density lipoprotein (HDL)-cholesterol (inverse normal transformed effect size), triglycerides (inverse normal transformed effect size) and diabetes, obtained from separate sample instrumental variable analysis in the Global Lipids Genetics Consortium Results^59^ and DIAGRAM^60^.

**Figure 5:**
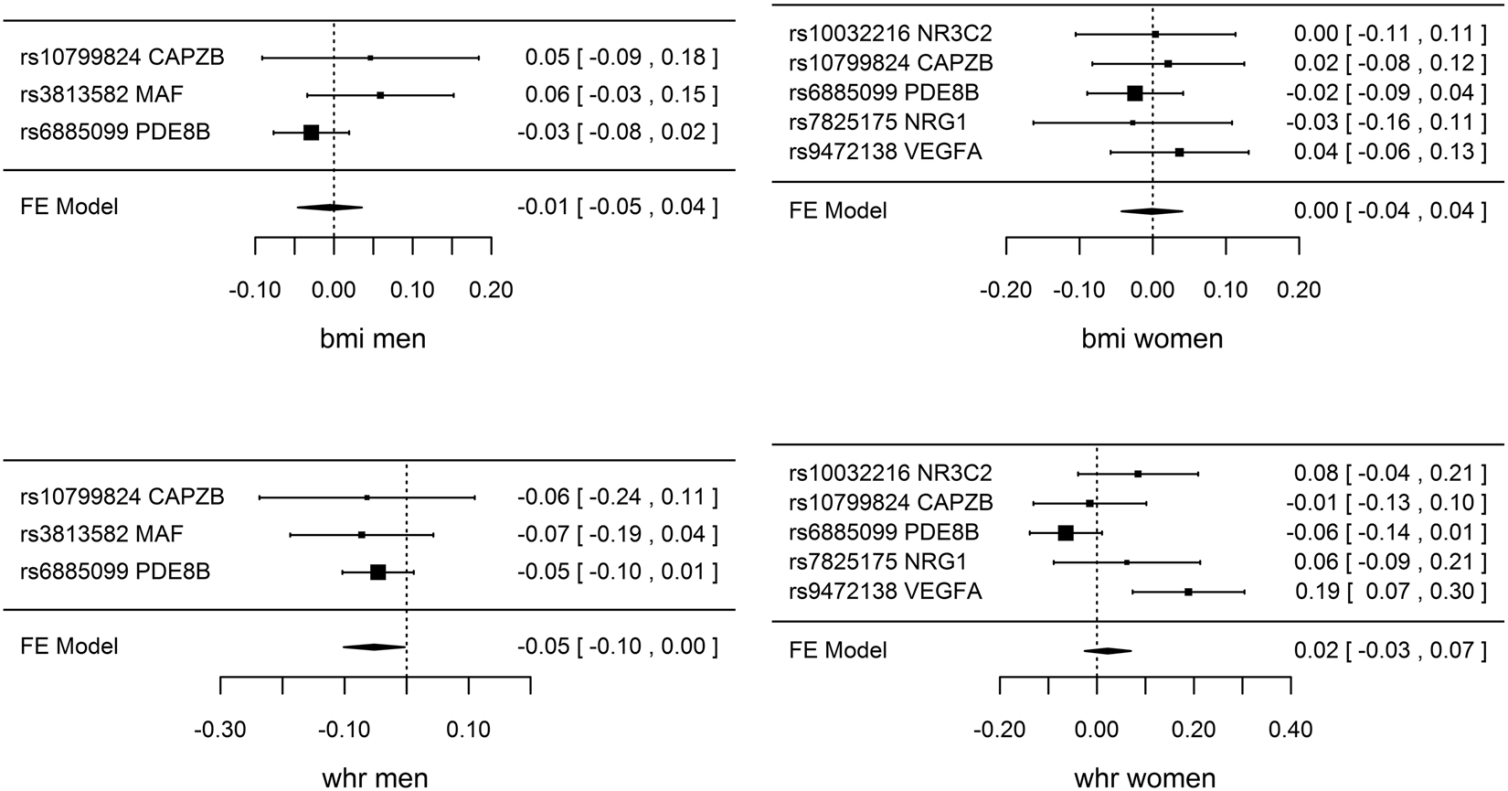
SNP specific and total associations per standard deviation (SD) thyroid-stimulating hormone (TSH) with body mass index (BMI) in men (inverse normal standard transformed) and in women (inverse normal standard transformed), waist-hip ratio (WHR) in men (inverse normal standard transformed) and in women (inverse normal standard transformed), obtained from separate sample instrumental variable analysis in GIANT^64, 65^.

**Figure 6:**
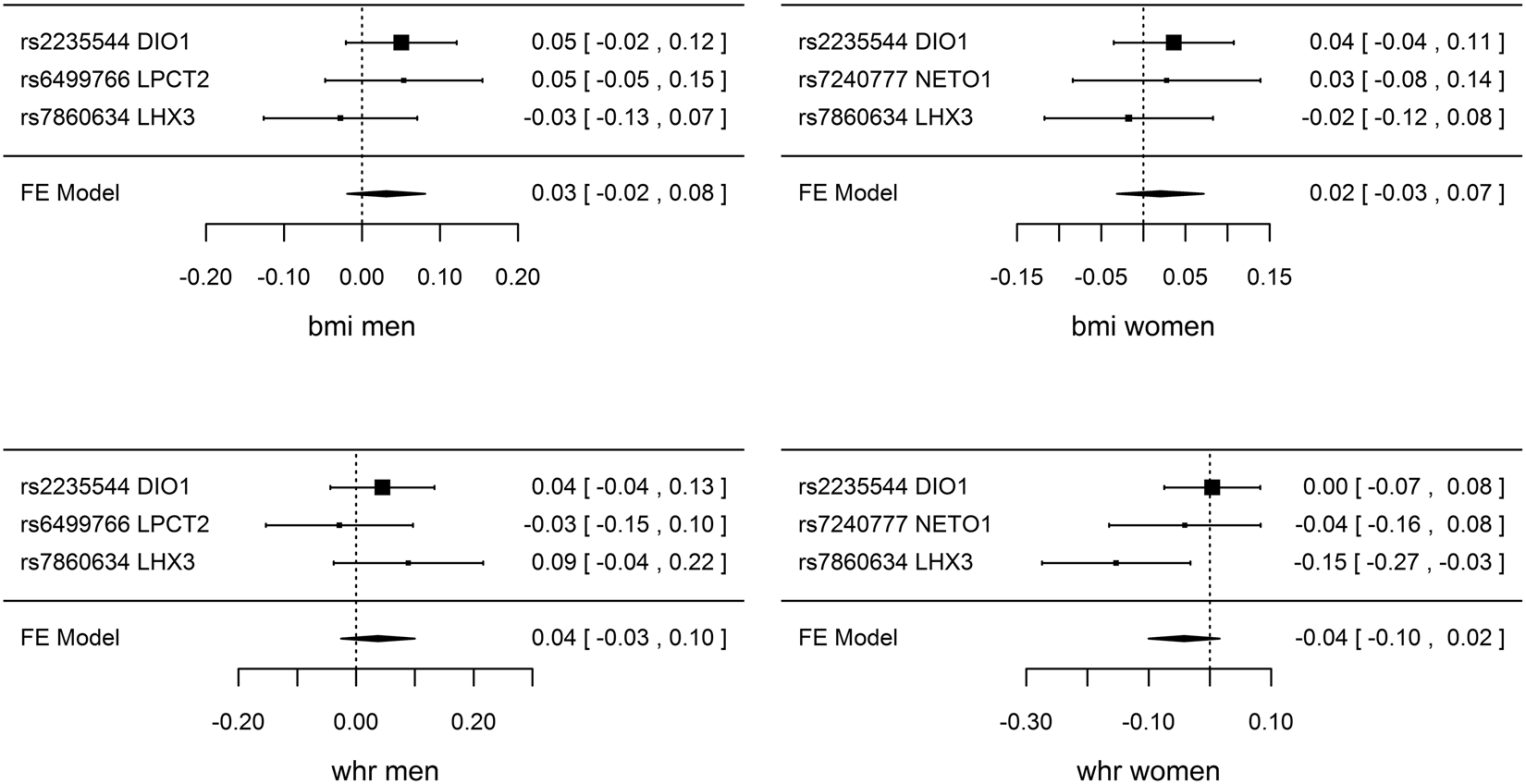
SNP specific and total associations per standard deviation (SD) free thyroxine (FT4) with body mass index (BMI) in men (inverse normal standard transformed) and in women (inverse normal standard transformed), waist-hip ratio (WHR) in men (inverse normal standard transformed) and in women (inverse normal standard transformed), obtained from separate sample instrumental variable analysis in GIANT^64, 65^.

Genetically predicted TSH, FT4 and TPOAb positivity were not associated with risk of CAD/MI when potentially pleiotropic SNPs were excluded (Table 1 and Fig. 1). The associations of TSH and TPOAb positivity with IHD and MI in CARDIoGRAMplusC4D 1000 Genomes and the association of TPOAb positivity with IHD in CARDIoGRAMplusC4D Metabochip showed heterogeneity when potentially pleiotropic SNPs were included (Table 1). Most of the associations with lipid profile (LDL-cholesterol, HDL-cholesterol or triglycerides) or diabetes (Figs 2 and 3, and Supplemental Tables 3–5) were null, but we identified an association of higher genetically predicted FT4 with lower LDL-cholesterol (Fig. 3 and Supplemental Table 4) and an association of genetically predicted TPOAb positivity with lower HDL-cholesterol (Fig. 4 and Supplemental Table 5). Genetically predicted TSH and FT4 were also unrelated to body mass index (BMI) and wasit-hip ratio (WHR) in sex-specific analysis, but genetically predicted higher TSH was nominally associated with lower WHR in men (Figs 5, 6 and Supplemental Tables 3 and 4). Most of the associations with glucose metabolism were null (Supplemental Figs 1, 2 and 3) but we identified an association of higher TSH with lower 2 hour glucose (Supplemental Fig. 1). These associations were generally robust to SNP selection, however, TPOAb positivity was associated with higher risk of CAD/MI when including the SNP with potential pleiotropy (Table 1), and the association of TSH with WHR in men became null when including the SNPs with potential pleiotropy (Supplemental Table 3). These associations were also generally robust to different statistical methods, however, the associations of TSH with WHR in men and 2 hour glucose in men and women became null when using a weighted median estimator or MR Egger, and there was an inverse association of TSH with HOMA-b using MR Egger (Supplemental Table 3). Taking into account multiple comparisons, all the associations, except for the inverse association of FT4 with LDL-cholesterol, were null after Bonferroni correction (corrected p-value: 0.05/5 (traits considered)/3 (exposures) = 0.003). The intercept from MR Egger for TSH and CAD/MI in CARDIoGRAMplusC4D Metabochip was smaller when including potentially pleiotropic SNPs, which might occur by chance, because we did not observe this in CARDIoGRAMplusC4D 1000 Genomes.

## Discussion

Using MR to obtain unconfounded estimates, genetically predicted TSH, FT4 and TPOAb positivity were not associated with risk of CAD/MI, despite a positive association of TPOAb positivity with CAD/MI in CARDIoGRAMplusC4D Metabochip when including a potentially pleiotropic SNP. Most of the associations with CVD risk factors were null, but genetically predicted higher TSH was associated with lower WHR in men and lower 2 hour glucose in men and women, genetically predicted higher FT4 was associated with lower LDL-cholesterol and genetically predicted TPOAb positivity was associated with lower HDL-cholesterol. This novel study does not corroborate a role of TSH, FT4 or TPOAb positivity in CAD/MI, although it cannot rule out a potential role in CVD risk factors.

To our knowledge, our study is the first MR examining the effects of TSH, FT4 and TPOAb positivity on IHD and CVD risk factors. Taking advantage of large publicly-available genome-wide association studies (GWAS) and large case-control studies with extensive genotyping enabled us to conduct a well-powered MR study in a cost-efficient way^54^. CARDIoGRAMplusC4D with 64,374 cases and 130,681 controls and CARDIoGRAMplusC4D 1000 Genomes with 60,801 cases and 123,504 controls has 0.8 power to detect an odds ratio (OR) of about 1.04 per standard deviation (SD) increase in TSH at an R^2^ of ~0.14 and an OR of about 1.07 per SD increase in FT4 at an R^2^ of ~0.04^55^. Our MR study design used separate samples, i.e., GWAS for the genetic associations with the exposures (TSH, FT4 and TPOAb positivity), and large case-control studies for the genetic associations with the outcomes. The samples were recruited from different studies, with a very small proportion (around 1–4%) of overlap between the GWAS for exposures^22, 47, 48^ and CARDIoGRAMplusC4D Metabochip and 1000 Genomes^50, 52^. As such, this MR is less open to confounding than one sample MR, because any correlation of the genetic variants with unmeasured confounders in the samples with TSH, FT4 and TPOAb positivity are less likely to be replicated in the samples with the outcomes, due to the different data structures^56^.

Nevertheless, several limitations exist. First, MR requires stringent assumptions, i.e., the genetic instruments are associated with the exposure, are not associated with the outcomes other than via the relevant exposure (no pleiotropy) and no confounders of the associations of the genetic instruments with the outcomes exist^45^. To satisfy these conditions, we only selected SNPs strongly associated with TSH, FT4 and TPOAb positivity. Most SNPs are from genes functionally relevant to thyroid function, such as *NFIA* which interacts with thyroid transcription factor 1, essential for thyroid-specific gene expression^47^. The selected SNPs are not known to affect CAD/MI other than via the relevant exposures (pleiotropy). However, the *DIO1* gene associated with FT4 is also associated with FT3 and FT3/FT4 ratio^57^. As such, we cannot exclude the role of other thyroid hormones. SNPs in the *ABO* gene are known to be strongly associated with IHD^52^, so we presented estimates excluding and including the *ABO* SNP rs657152 for TSH. Sensitivity analyses using a weighted median method and MR Egger with different assumptions from inverse variance weighting (IVW) also provided consistent results for most associations, which adds some strength. Second, use of separate samples means that we cannot test the possibility that the SNPs for TSH and FT4 have different effects on CAD/MI at different levels of the exposures or at different ages. Generally causal factors are consistent, although interventions may not always have consistent effects, because the distribution of causal factors may vary across populations. Third, population stratification might affect the MR estimates. However, the genetic associations with TSH, FT4 and TPOAb positivity and with CAD/MI and CVD risk factors are all from studies conducted in Caucasians, largely of European descent, with genomic control^50, 52, 58–67^. Fourth, our study was limited to Caucasians and might not apply to other populations. However, the effects of causal factors are not expected to vary by setting^68^. The effect of TSH-lowering intervention on lipid profile is consistent across countries^36–38^. Fifth, the influence of genetic determinants might be damped or buffered by compensatory developmental processes, i.e., canalization^45^. However, the GWAS for all exposures were conducted in people with normal thyroid function^47^. Sixth, we cannot determine the clinical significance of these small effect sizes, because MR estimates should be interpreted as hypothesis testing for causation, rather than indicating the exact size of causal effects^69^. However, small effects that may not be clinically significant may still be an important determinant of population health. Seventh, blood pressure was not included in the study, because the publicly available data providing the genetic association with blood pressure, i.e., the International Consortium for Blood pressure^70, 71^, does not give the direction of association. Eighth, associations with WHR in men and with 2 hour glucose in men and women were null after Bonferroni correction. However, most outcomes were expected to have no association with TSH, so the value of adjusting for multiple comparisons is moot. Given the limited SNPs available for sex-specific analyses of adiposity, the associations with adiposity need to be replicated in other MR studies using different genetic determinants. Ninth, MR requires large sample sizes, because the genetic predictors identified in GWAS only explain a small proportion of the variance in thyroid function^47^. The genetic architecture for thyroid function is also unclear, and GWAS based on statistical analysis may not identify all functionally relevant genetic variants. A large proportion of the heritability in thyroid function is still unexplained^49^. There were also a limited number of SNPs for FT4 and TPOAb positivity, and for TSH in sex-specific analyses. Tenth, non-collapsibility and ascertainment bias are often encountered in MR when using a binary outcome from case-control studies, and the estimate from MR Egger provides an approximation^72^. Lastly, we could not assess whether the effects on IHD and most CVD risk factors vary by sex. Sex-specific associations with IHD, lipids, diabetes and glucose metabolism are unavailable. As such our estimates for these outcomes are likely to be conservative as some associations could be sex-specific.

Our study is inconsistent with some observational studies showing TSH associated with higher risk of CVD events^34^. The null associations of TSH and FT4 with most CVD risk factors are also inconsistent with some studies showing higher TSH and lower FT4 associated with an unhealthier CVD risk profile^24–27^. However, observational studies are open to residual confounding and reverse causality, making them difficult to use as a basis for action^73^. Moreover, the hypothalamic–pituitary–thyroid axis interacts with the hypothalamic–pituitary–gonadal axis^16^, and might be affected by diet^74^, so separating their roles is challenging in observational studies. The association of FT4 with lower LDL-cholesterol is consistent with some RCTs showing beneficial effect of levothyroxine, a manufactured form of thyroxine, on LDL-cholesterol^36–38^. The Coronary Drug Project found a detrimental effect of dextrothyroxine on CVD^40^. However, given the pleiotropic effects of dextrothyroxine^75^, whether the effect is mediated by lowering TSH is unclear. The effect of levothyroxine on CVD, is being examined in trials but results are unavailable^43, 44^.

Several explanations might exist for the largely null associations of TSH, FT4 and TPOAb positivity with IHD and CVD risk factors. First, the genetic variants may not be determinants of these exposures. The SNPs are from GWAS, which are selected based on statistical correlation rather than biological pathways. The genetic variants for TSH and FT4 may also be incomplete; because SNPs in the functionally relevant genes, such as *TSHB* and *TSHR*, were not identified by GWAS. The GWAS may also be underpowered to detect a convincing correlation between TSH and FT4 variants^47^; only the *FOXE* gene for TSH is also associated with FT4 (Supplemental Table 1). Replication using more comprehensive genetic variants is needed. Second, thyroid hormones might not affect IHD, or have little effect on IHD, although they may affect lipid profile. Consistently, several drugs, such as estrogen, niacin and fibrates, generally modulate lipids in the desired direction but failed to reduce CVD^76^. Third, given that sex-specific analysis for CAD/MI is not available, we cannot rule out positive estimates in sex-specific analyses.

From the perspective of clinical practice, MR could have helped avoid several very expensive late-stage clinical trial failures and might improve prediction of what RCTs will show^77^. Our study adds to the limited evidence concerning the role of TSH, FT4 and TPOAb positivity in CAD/MI and provides little indication that TSH, FT4 or TPOAb positivity affects the risk of CAD/MI, although we cannot rule out their potential effects on CVD risk factors. Our findings do not corroborate thyroid function as targets of intervention for IHD. Replication using more comprehensive genetic variants in sex-specific analysis is required.

## Conclusions

This novel study provides little indication that TSH, FT4 or TPOAb positivity affects IHD, despite potential effects on CVD risk factors. Our findings do not corroborate thyroid function as targets of intervention for IHD. Although we cannot completely rule out an effect of thyroid function on IHD, our study suggests other targets of intervention should be sought to tackle the leading cause of global morbidity and mortality.

## Methods

Genetic predictors, i.e., SNPs, for TSH and FT4, were obtained from a large meta-analysis of GWAS (n = 26,420 for TSH and n = 17,520 for FT4) of European descent from 15 cohorts (mean age 42.5 to 79.0 years old), which excluded people taking thyroid medication, with thyroid surgery, or with TSH values outside the normal range (TSH > 4.0 mIU/L or TSH < 0.4 mIU/L), with genetic associations adjusted for age, age-squared, and sex^47^, and a large meta-analysis of whole-genome sequence-based analysis (n = 16,335) in healthy individuals of European descent from 7 cohorts (mean age 7.5 to 54.1 years old), with genetic associations adjusted for age, age-squared, sex and any other cohort-specific variables^49^. Genetic predictors for TSH were also obtained from a large whole-genome sequencing study of the Icelandic population (n = 2,636), mean age 55 years old, with genetic associations adjusted for age and sex^48^. Genetic predictors for TPOAb positivity were obtained from a large meta-analysis of GWAS in general population in 18,297 Caucasians from 11 studies (mean age 46.9 to 74.8 years old), with genetic associations adjusted for age and sex^22^. Only SNPs strongly (p-value < 5 × 10^−8^) and solely associated with TSH or FT4 were used. Correlations between the selected SNPs, i.e., LD, were checked using a comprehensive genetic cross-reference system, Ensembl (http://www.ensembl.org/index.html). Where SNPs were highly correlated (r^2^ > 0.7), the SNP with the larger p-value was discarded. Less strongly correlated SNPS were included taking LD into account. Pleiotropy, i.e., associations with CAD/MI other than via the relevant exposures were checked using two comprehensive genetic cross-reference systems, Ensembl (http://www.ensembl.org/index.html) and MR Catalogue (http://mrcatalogue.medschl.cam.ac.uk/), which provide well-established effects of known SNPs in the form of cross-references from SNP to trait/phenotype.

Data on CAD/MI have been contributed by CARDIoGRAMPLUSC4D investigators and downloaded from www.CARDIOGRAMPLUSC4D.ORG. CARDIoGRAMplusC4D Metabochip is a large case (n = 63,746)-control (n = 130,681) study, largely of European descent, mean age 56.9 years old, with genetic associations adjusted for age, sex and study specific covariates^58^. CARDIOGRAM is a subset of CARDIoGRAMplusC4D with more extensive genotyping^52^. Genetic associations with CAD/MI were obtained from CARDIoGRAMplusC4D if available; or else from CARDIOGRAM. CARDIoGRAMplusC4D 1000 Genomes is a case (n = 60,801)-control (n = 123,504) study, 77% of European descent, which partly overlaps with CARDIoGRAMplusC4D Metabochip^50^. Genetic associations with lipids (as inverse normal transformed effect sizes), including HDL-cholesterol, LDL-cholesterol and triglycerides, adjusted for age, age^2^ and sex, were obtained from the Global Lipids Genetics Consortium Results (http://csg.sph.umich.edu//abecasis/public/lipids2013/), with 188,577 participants of European descent and 7,898 participants of non-European descent, mean age 55.2 years old^59^. Genetic associations with diabetes, adjusted for age and sex, were obtained from the DIAbetes Genetics Replication And Meta-analysis (DIAGRAM), http://diagram-consortium.org/downloads.html, case (n = 34,840)-control (n = 114,981) study, mean age 57.4 years old^60^. Genetic associations, adjusted for age, sex and study-specific covariates, with HbA_1c_ (n = 46,368)^61^, fasting glucose (mmol/L) (n = 108,557)^62^, 2 hour glucose (mmol/L) adjusted for BMI (n = 42,854)^62^, log-transformed fasting insulin (pmol/L) (n = 108,557)^62^, β-cell function (log-transformed homeostatic model assessment (HOMA-b), n = 46,186)^63^ and HOMA-insulin resistance (IR) (log-transformed HOMA-IR, n = 46,186)^63^ were obtained from the Meta-Analyses of Glucose and Insulin-related traits Consortium (MAGIC), http://www.magicinvestigators.org/, of people with no diabetes and of European ancestry. Genetic associations, adjusted for age, age^2^, and study-specific covariates, with inverse standard normal transformed BMI in 152,893 men and 171,977 women of European ancestry^64^ and inverse standard normal transformed WHR in 93,480 men and 116,741 women^65^, were obtained from the Genetic Investigation of ANthropometric Traits (GIANT), https://www.broadinstitute.org/collaboration/giant/index.php/GIANT_consortium_data_files.

The associations of TSH, FT4 and TPOAb positivity with CAD/MI and CVD risk factors were obtained from separate sample instrumental variable (SSIV) using the Wald estimate (the ratio of genetic association with CAD/MI and with TSH, FT4 and TPOAb positivity).

SNP-specific estimates were then combined using IVW with fixed effects for uncorrelated SNP and weighted generalized linear regression for correlated SNPs^54^. The standard deviation of the ratio was obtained from Fieller’s theorem^78^. The instrument strength is typically represented by the F-statistic^72^. In the two-sample summary data context and with uncorrelated genetic variants, we approximated the F-statistic for each genetic variant using an established formula^72^, and calculated the mean F-statistic for TSH, FT4 and TPOAb positivity. The IVW method assumes that all genetic variants are valid instruments^72^. We also used a weighted median method and MR Egger, because we wanted to account for the potential bias from invalid instruments when using multiple genetic variants as instrument^79^. The weighted median method provides a consistent estimate of the causal effect even when up to 50% of the information contributing to the analysis comes from genetic variants that are invalid instruments^80^. MR Egger tests for directional pleiotropy, under the assumption that pleiotropic effects of genetic variants are independent of instrumental strength^80^. To examine potential bias from SNPs which had associations with the outcomes other than via these exposures (violation of the exclusion restriction assumption), sensitivity analyses including SNPs which had potentially pleiotropic associations with the outcomes were conducted. We tested for heterogeneity using Cochran’s Q test, and tested for directional pleiotropy by testing the intercept from MR Egger, where a p-value of <0.05 indicates the presence of directional pleiotropy across the genetic variants included in the analyses^81^. To account for multiple comparisons, we also used a Bonferroni correction. All statistical analyses were conducted using R version 3.3.2 (R Foundation for Statistical Computing, Vienna, Austria).

### Data availability statement

Data on CAD/MI have been contributed by CARDIoGRAMPLUSC4D investigators and downloaded from www.CARDIOGRAMPLUSC4D.ORG. Genetic associations with lipids were obtained from the Global Lipids Genetics Consortium Results (http://csg.sph.umich.edu//abecasis/public/lipids2013/). Genetic associations with diabetes are from the DIAbetes Genetics Replication And Meta-analysis (DIAGRAM), http://diagram-consortium.org/downloads.html. Genetic associations with glucose metabolism indicators were obtained from the Meta-Analyses of Glucose and Insulin-related traits Consortium (MAGIC), http://www.magicinvestigators.org/. Genetic associations with inverse standard normal transformed BMI and inverse standard normal transformed WHR were obtained from the Genetic Investigation of ANthropometric Traits (GIANT), https://www.broadinstitute.org/collaboration/giant/index.php/GIANT_consortium_data_files.

### Ethical approval

This analysis of publicly available data does not require ethical approval.

## Electronic supplementary material

Supplementary information
