# Supplementary material for: Thyroid function and ischemic heart disease: a Mendelian randomization study
Source: Sci Rep. 2017 Aug 17;7:8515. doi: 10.1038/s41598-017-07592-z (PMC5561103; doi:10.1038/s41598-017-07592-z)
Supplement: Supplementary file 1 — Supplementary information [file 41598_2017_7592_MOESM1_ESM.pdf]

Thyroid function and ischemic heart disease: a Mendelian randomization study

Jie V Zhao, C Mary Schooling

Supplemental Material

Supplemental Table 1: Associations of genetic determinants with thyroid-stimulating hormone (TSH), free thyroxine (FT4) and thyroid peroxidase antibody (TPOAb) positivity in men and women

Exposure	SNPs	Nearby gene	Effect allele	beta	SE	P value	Potential pleiotropy	Note
TSH	rs6885099	*PDE8B*	A	-0.14	0.01	1.95×10^{-56}		
	rs10799824	*CAPZB*	A	-0.11	0.01	3.60×10^{-21}		
	rs3813582	*MAF*	T	0.08	0.01	8.45×10^{-18}		
	rs11755845	*VEGFA*	T	-0.07	0.01	1.68×10^{-10}		
	rs10032216	*NR3C2*	T	0.09	0.01	9.28×10^{-16}		
	rs334699	*NFIA*	A	-0.14	0.02	5.40×10^{-12}		
	rs4804416	*INSR*	T	-0.06	0.01	3.16×10^{-10}		
	rs1537424	*MBIP*	T	-0.05	0.01	1.17×10^{-8}		
	rs11624776	*ITPK1*	A	-0.06	0.01	1.79×10^{-9}		
	rs7825175	*NRG1*	A	-0.07	0.01	2.94×10^{-9}		
	rs13015993	*IGFBP5*	A	0.08	0.01	3.24×10^{-15}	MI and childhood obesity[1,2]	
	rs753760	*PDE10A*	C	0.10	0.01	1.21×10^{-24}	waist circumstance[3,4]	
	rs9915657	*SOX9*	T	-0.06	0.01	7.53×10^{-13}	BMI[5]	
	rs9472138	*VEGFA*	T	-0.08	0.01	6.72×10^{-16}	type 2 diabetes[6]	
	rs10519227	*FGF7*	A	-0.07	0.01	1.02×10^{-11}	Waist hip ratio[5] triglycerides[7]	
	rs17723470	*PRDM11*	T	-0.07	0.01	8.83×10^{-11}	obesity[5]	
	rs17776563	*MIR1179*	A	-0.06	0.01	2.89×10^{-10}	LDL-cholesterol[8]	
	rs9497965	*SASH1*	T	0.05	0.01	2.25×10^{-8}	BMI and blood pressure[4,9]	
	rs1571583	*GLIS3*	A	0.06	0.01	2.55×10^{-8}	type 2 diabetes, obesity and education[5,6,10]	
	rs657152	*ABO*	A	0.06	0.01	4.11×10^{-10}	CAD/MI	unavailable in the Global Lipids Genetics Consortium Results, no proxy SNP
	rs7568039	*IGFBP2*	A	-0.12	0.01	2.11×10^{-19}	CAD/MI[1]	unavailable in CARDIoGRAMplusC4D Metabochip
	rs310763	*SYN2*	T	0.08	0.01	6.15×10^{-9}		
	rs2928167	*PDE8B*	G	-0.15	0.02	5.94×10^{-14}		unavailable in CARDIoGRAMplusC4D Metabochip, rs12523579 was used as a proxy
	rs112817873	*FOXE1*	T	-0.14	0.02	6.15×10^{-20}		unavailable in CARDIoGRAMplusC4D Metabochip and 1000 Genomes
	rs116909374	*MBIP*	T	-0.21	0.03	4.69×10^{-11}		unavailable in CARDIoGRAMplusC4D Metabochip
	rs139242164	*GNAS*	T	0.30	0.04	1.3×10^{-12}		unavailable in CARDIoGRAMplusC4D Metabochip
FT4	rs2235544	*DIO1*	A	0.14	0.01	7.87×10^{-32}		
	rs7860634	*LHX3*	A	0.10	0.01	2.30×10^{-14}		
	rs7045138	*FOXE1*	T	0.10	0.02	1.50×10^{-11}	HDL[8]	unavailable in CARDIoGRAMplusC4D 1000 Genomes and the Global Lipids

								Genetics Consortium Results; rs4297160 was used as a proxy
	rs11726248	*AADAT*	A	0.11	0.02	5.20×10^{-9}	LDL[7]	
	rs113107469	*B4GALT6*	T	0.22	0.04	1.27×10^{-9}		
TPOAb	rs11675434	*TPO*	T	0.08	0.01	1.5×10^{-16}		
	rs10944479	*BACH2*	A	0.10	0.02	9.9×10^{-10}		
	rs653178	*ATXN2*	C	0.06	0.01	4.0×10^{-8}	MI[1] and blood pressure[11]	

SE, standard error; TSH, thyroid-stimulating hormone; FT4, free thyroxine; TPOAb, thyroid peroxidase antibody;

Supplemental Table 2: Sex-specific associations of selected genetic determinants with thyroid-stimulating hormone (TSH) and free thyroxine (FT4)

Exposure	SNPs	Nearby gene	Effect allele	Men			Women		
				**beta**	**SE**	**P value**	**beta**	**SE**	**P value**
TSH	rs6885099	*PDE8B*	A	-0.17	0.01	2.70×10^{-38}	-0.12	0.01	6.09×10^{-24}
	rs10799824	*CAPZB*	A	-0.10	0.02	1.17×10^{-8}	-0.12	0.02	2.69×10^{-14}
	rs3813582	*MAF*	T	0.12	0.01	6.17×10^{-17}			
	rs10032216	*NR3C2*	T				0.11	0.01	1.72×10^{-13}
	rs7825175	*NRG1*	A				-0.08	0.02	1.64×10^{-8}
	rs9472138	*VEGFA*	T				-0.09	0.01	6.30×10^{-12}
FT4	rs2235544	*DIO1*	A	0.14	0.02	4.59×10^{-15}	0.13	0.02	2.62×10^{-8}
	rs7860634	*LHX3*	A	0.11	0.02	1.72×10^{-8}	0.10	0.02	5.01×10^{-8}
	rs6499766	*LPCT2*	A	0.10	0.02	4.63×10^{-8}			
	rs7240777	*NETO1*	A				-0.08	0.02	3.49×10^{-8}

SE, standard error; TSH, thyroid-stimulating hormone; FT4, free thyroxine

Supplemental Table 3: Associations per standard deviation (SD) thyroid-stimulating hormone (TSH) with diabetes, lipids, glycemic traits and adiposity, obtained from separate sample instrumental variable analysis in DIAGRAM,[12] the Global Lipids Genetics Consortium Results,[8] MAGIC,[3,13,14] and GIANT,[15,16] using different SNP selection and methods

Outcome		SNPs without pleiotropy*					All SNPs			
		No. of SNPs	Methods	OR	95% CI	Intercept p value†	No. of SNPs	OR	95% CI	Intercept p value†
**Diabetes**		13	IVW	0.97	0.85 to 1.11		21	0.93	0.84 to 1.04	
			WM	0.88	0.73 to 1.07			0.87	0.74 to 1.02	
			MR Egger	1.00	0.88 to 1.14	0.001		0.98	0.88 to 1.10	2.0×10^{-6}
				β	95% CI			β	95% CI	
**Lipids**	LDL-cholesterol (effect size)	18	IVW	0.02	-0.01 to 0.05		21	0.03	-0.002 to 0.05	
			WM	0.03	-0.02 to 0.07			0.03	-0.02 to 0.07	
			MR Egger	0.01	-0.02 to 0.04	0.01		0.01	-0.02 to 0.04	0.0003
	HDL-cholesterol (effect size)	18	IVW	0.004	-0.02 to 0.03		21	0.001	-0.02 to 0.03	
			WM	0.03	-0.01 to 0.06			0.01	-0.03 to 0.05	
			MR Egger	-0.01	-0.04 to 0.02	1.8×10^{-5}		-0.01	-0.04 to 0.02	9.6×10^{-6}
	Triglycerides (effect size)	18	IVW	0.003	-0.02 to 0.03		21	0.01	-0.02 to 0.03	
			WM	0.002	-0.03 to 0.04			0.005	-0.03 to 0.04	
			MR Egger	-0.001	-0.03 to 0.02	1.6×10^{-5}		-0.001	-0.03 to 0.02	4.1×10^{-6}
**Glycemic traits**	Fasting glucose	13	IVW	-0.004	-0.03 to 0.02		21	-0.002	-0.03 to 0.02	
			WM	-0.01	-0.05 to 0.03			-0.007	-0.04 to 0.03	
			MR Egger	-0.004	-0.04 to 0.03	0.94		0.001	-0.03 to 0.03	0.29
	HbA$_{1c}$%	13	IVW	0.005	-0.02 to 0.03		21	0.01	-0.01 to 0.03	
			WM	0.007	-0.03 to 0.04			0.005	-0.02 to 0.03	
			MR Egger	0.01	-0.02 to 0.04	0.20		0.02	-0.01 to 0.04	0.06
	HOMA-IR (log transformed)	13	IVW	-0.02	-0.05 to 0.01		21	-0.01	-0.03 to 0.02	
			WM	-0.005	-0.05 to 0.04			-0.003	-0.04 to 0.04	
			MR Egger	-0.03	-0.06 to 0.01	0.11		-0.01	-0.04 to 0.02	0.18
	HOMA-b (log transformed)	13	IVW	-0.02	-0.05 to 0.004		21	-0.01	-0.03 to 0.01	
			WM	-0.01	-0.05 to 0.02			-0.01	-0.04 to 0.03	
			MR Egger	**-0.03**	**-0.05 to -0.002**	0.09		-0.02	-0.04 to 0.01	0.02
	Fasting insulin (log transformed)	13	IVW	-0.02	-0.05 to 0.004		21	-0.01	-0.04 to 0.01	
			WM	-0.03	-0.07 to 0.01			-0.01	-0.05 to 0.02	
			MR Egger	-0.03	-0.06 to 0.004	0.39		-0.02	-0.04 to 0.01	0.49
	2hr glucose adjusted for BMI	13	IVW	**-0.18**	**-0.32 to -0.04**		21	**-0.16**	**-0.28 to -0.04**	
			WM	-0.20	-0.41 to 0.01			**-0.18**	**-0.37 to -0.001**	
			MR Egger	-0.17	-0.36 to 0.02	0.77		**-0.16**	**-0.29 to -0.03**	0.81
**Adiposity**	BMI men (effect size)	3	IVW	-0.01	-0.05 to 0.04		6	0.01	-0.03 to 0.04	
			WM	-0.002	-0.05 to 0.04			0.001	-0.04 to 0.04	
			MR Egger	-0.17	-0.36 to 0.02	0.08		0.01	-0.03 to 0.05	0.52
	BMI women (effect size)	5	IVW	-0.001	-0.04 to 0.04		7	0.001	-0.04 to 0.04	
			WM	-0.003	-0.05 to 0.05			0.004	-0.04 to 0.05	
			MR Egger	-0.001	-0.04 to 0.04	0.59		-0.002	-0.04 to 0.04	0.55
	WHR men (effect size)	3	IVW	**-0.05**	**-0.10 to -0.003**		6	-0.02	-0.06 to 0.02	
			WM	-0.05	-0.10 to 0.001			-0.05	-0.10 to 0.005	
			MR Egger	-0.01	-0.24 to 0.23	0.70		0.02	-0.03 to 0.07	0.001
	WHR women (effect size)	5	IVW	0.02	-0.03 to 0.07		7	0.02	-0.02 to 0.06	
			WM	-0.0002	-0.07 to 0.07			-0.002	-0.07 to 0.07	
			MR Egger	0.03	-0.02 to 0.07	0.0002		0.01	-0.04 to 0.07	0.01

BMI, body mass index; OR, odds ratio; CI, confidence interval; HDL, high-density lipoprotein; LDL, low-density lipoprotein; IVW, inverse variance weighting; WM, weighted median method.

[*]SNPs with potential pleiotropy for lipids: rs13015993, rs657152, rs10519227, rs17776563; SNPs with potential pleiotropy for diabetes and glycemic traits: rs13015993, rs657152, rs9472138, rs9915657, rs10519227, rs17723470, rs9497965 and rs1571583; SNPs with potential pleiotropy for adiposity: rs13015993, rs753760, and rs10519227 in men; rs753760 and rs9497965 in women

[†] The intercept can be interpreted as an estimate of the average pleiotropic effect across the genetic variants where a corresponding p-value of < 0.05 indicates the presence of directional pleiotropy across the genetic variants included in the analyses.

Supplemental Table 4: Associations per standard deviation (SD) free thyroxine (FT4) with diabetes, lipids, glycemic traits and adiposity, obtained from separate sample instrumental variable analysis in DIAGRAM,[12] the Global Lipids Genetics Consortium Results,[8] MAGIC,[3,13,14] and GIANT,[15,16] using different SNP selection and methods

Outcome		SNPs without pleiotropy*					All SNPs‡			
		No. of SNPs	Methods	OR	95% CI	Intercept p value†	No. of SNPs	OR	95% CI	Intercept p value†
**Diabetes**		4	IVW	0.94	0.79 to 1.10		4	0.94	0.79 to 1.10	
			WM	0.93	0.77 to 1.11			0.93	0.77 to 1.11	
			MR Egger	0.99	0.81 to 1.22	0.36		0.99	0.81 to 1.22	0.36
				β	95% CI			β	95% CI	
**Lipids**	LDL-cholesterol (effect size)	2	IVW	**-0.09**	**-0.16 to -0.03**		4	**-0.07**	**-0.12 to -0.02**	
			WM	**-0.09**	**-0.16 to -0.03**			**-0.09**	**-0.15 to -0.03**	
			MR Egger	NA	NA	NA		-0.03	-0.09 to 0.02	0.006
	HDL-cholesterol (effect size)	2	IVW	0.01	-0.05 to 0.07		4	-0.01	-0.05 to 0.04	
			WM	0.01	-0.05 to 0.07			0.003	-0.05 to 0.06	
			MR Egger	NA	NA	NA		0.003	-0.05 to 0.05	0.10
	Triglycerides (effect size)	2	IVW	-0.01	-0.07 to 0.04		4	-0.01	-0.05 to 0.04	
			WM	-0.01	-0.07 to 0.04			-0.01	-0.06 to 0.05	
			MR Egger	NA	NA	NA		0.01	-0.04 to 0.06	0.10
**Glycemic traits**	Fasting glucose	4	IVW	0.002	-0.03 to 0.04		4	0.002	-0.03 to 0.04	
			WM	-0.02	-0.06 to 0.02			-0.02	-0.06 to 0.02	
			MR Egger	-0.15	-0.47 to 0.18	0.36		-0.15	-0.47 to 0.18	0.36
	HbA_{1c}%	4	IVW	-0.01	-0.04 to 0.02		4	-0.01	-0.04 to 0.02	
			WM	-0.006	-0.04 to 0.03			-0.006	-0.04 to 0.03	
			MR Egger	0.008	-0.21 to 0.23	0.88		0.008	-0.21 to 0.23	0.88
	HOMA-IR (log transformed)	4	IVW	0.01	-0.03 to 0.05		4	0.01	-0.03 to 0.05	
			WM	0.02	-0.03 to 0.06			0.02	-0.03 to 0.06	
			MR Egger	0.06	-0.20 to 0.32	0.71		0.06	-0.20 to 0.32	0.71
	HOMA-b (log transformed)	4	IVW	0.01	-0.02 to 0.04		4	0.01	-0.02 to 0.04	
			WM	0.005	-0.03 to 0.04			0.005	-0.03 to 0.04	
			MR Egger	0.12	-0.09 to 0.33	0.30		0.12	-0.09 to 0.33	0.30
	Fasting insulin (log transformed)	4	IVW	0.02	-0.02 to 0.05		4	0.02	-0.02 to 0.05	
			WM	0.02	-0.02 to 0.07			0.02	-0.02 to 0.07	
			MR Egger	0.10	-0.14 to 0.35	0.48		0.10	-0.14 to 0.35	0.48
	2hr glucose adjusted for BMI	4	IVW	-0.07	-0.25 to 0.11		4	-0.07	-0.25 to 0.11	
			WM	-0.08	-0.29 to 0.13			-0.08	-0.29 to 0.13	
			MR Egger	-0.62	-1.81 to 0.58	0.36		-0.62	-1.81 to 0.58	0.36
**Adiposity**	BMI men (effect size)	3	IVW	0.03	-0.02 to 0.08		3	0.03	-0.02 to 0.08	
			WM	0.05	-0.01 to 0.11			0.05	-0.01 to 0.11	
			MR Egger	0.11	-0.28 to 0.49	0.70		0.11	-0.28 to 0.49	0.70
	BMI women (effect size)	3	IVW	0.02	-0.03 to 0.07		3	0.02	-0.03 to 0.07	
			WM	0.03	-0.02 to 0.09			0.03	-0.02 to 0.09	
			MR Egger	0.02	-0.03 to 0.08	0.80		0.02	-0.03 to 0.08	0.80
	WHR men (effect size)	3	IVW	0.04	-0.03 to 0.10		3	0.04	-0.03 to 0.10	
			WM	0.05	-0.03 to 0.12			0.05	-0.03 to 0.12	
			MR Egger	0.13	-0.34 to 0.60	0.69		0.13	-0.34 to 0.60	0.69
	WHR women (effect size)	3	IVW	-0.04	-0.10 to 0.02		3	-0.04	-0.10 to 0.02	
			WM	-0.03	-0.09 to 0.04			-0.03	-0.09 to 0.04	
			MR Egger	-0.04	-0.19 to 0.10	0.85		-0.04	-0.19 to 0.10	0.85

BMI, body mass index; OR, odds ratio; CI, confidence interval; HDL, high-density lipoprotein; LDL, low-density lipoprotein; IVW, inverse variance weighting; NA, not available; WM, weighted median method.

[*]SNPs with potential pleiotropy for lipids: rs7045138 and rs11726248; no SNPs with potential pleiotropy for diabetes, glycemic traits or adiposity.

[†]The intercept can be interpreted as an estimate of the average pleiotropic effect across the genetic variants where a corresponding p-value of <0.05 indicates the presence of directional pleiotropy across the genetic variants included in the analyses.

[‡] Rs4297160 was used as a proxy for rs7045138 in the Global Lipids Genetics Consortium Results; rs113107469 was not available in the Global Lipids Genetics Consortium Results

Supplemental Table 5: Associations of thyroid peroxidase antibody (TPOAb) positivity with diabetes, lipids, and glycemic traits, obtained from separate sample instrumental variable analysis in DIAGRAM,[12] the Global Lipids Genetics Consortium Results,[8] and MAGIC,[3,13,14] using different methods

**Outcome**		No. of SNPs	Methods	OR	95% CI	Intercept p value†
**Diabetes**		3	IVW	1.07	0.82 to 1.39	0.72
			WM	1.07	0.79 to 1.45	
			MR Egger	0.85	0.24 to 3.08	
				β	95% CI	
**Lipids**	LDL-cholesterol (effect size)	3	IVW	-0.08	-0.16 to 0.01	
			WM	-0.08	-0.20 to 0.05	
			MR Egger	-0.02	-0.36 to 0.33	0.28
	HDL-cholesterol (effect size)	3	IVW	**-0.09**	**-0.17 to -0.01**	
			WM	-0.07	-0.20 to 0.07	
			MR Egger	-0.11	-0.34 to 0.12	0.09
	Triglycerides (effect size)	3	IVW	0.05	-0.02 to 0.13	
			WM	0.09	-0.02 to 0.19	
			MR Egger	0.02	-0.05 to 0.10	0.003
**Glycemic traits**	Fasting glucose	3	IVW	0.05	-0.01 to 0.11	
			WM	0.07	-0.01 to 0.14	
			MR Egger	0.05	-0.08 to 0.18	0.94
	HbA_{1c}%	3	IVW	-0.03	-0.09 to 0.03	
			WM	-0.002	-0.07 to 0.07	
			MR Egger	**-0.09**	**-0.15 to -0.03**	0.0004
	HOMA-IR (log transformed)	3	IVW	0.04	-0.03 to 0.10	
			WM	0.03	-0.04 to 0.11	
			MR Egger	0.04	-0.03 to 0.12	0.61
	HOMA-b (log transformed)	3	IVW	-0.02	-0.07 to 0.04	
			WM	0.001	-0.07 to 0.07	
			MR Egger	-0.01	-0.11 to 0.10	0.53
	Fasting insulin (log transformed)	3	IVW	0.04	-0.03 to 0.10	
			WM	0.04	-0.03 to 0.11	
			MR Egger	0.04	-0.03 to 0.11	0.96
	2hr glucose adjusted for BMI	3	IVW	0.08	-0.24 to 0.40	
			WM	0.06	-0.33 to 0.46	
			MR Egger	0.18	-0.19 to 0.54	0.18

BMI, body mass index; OR, odds ratio; CI, confidence interval; HDL, high-density lipoprotein; LDL, low-density lipoprotein; IVW, inverse variance weighting; WM, weighted median method; TPOAb, thyroid peroxidase antibody;

†The intercept can be interpreted as an estimate of the average pleiotropic effect across the genetic variants where a corresponding p-value of < 0.05 indicates the presence of directional pleiotropy across the genetic variants included in the analyses.

Supplemental Figure 1: SNP specific and total associations per standard deviation (SD) thyroid-stimulating hormone (TSH) with fasting glucose, hbA1c, log-transformed HOMA-IR, log transformed HOMA-b, log transformed fasting insulin and 2 hour glucose adjusted for body mass index (BMI), obtained from separate sample instrumental variable analysis in MAGIC.[3,13,14]

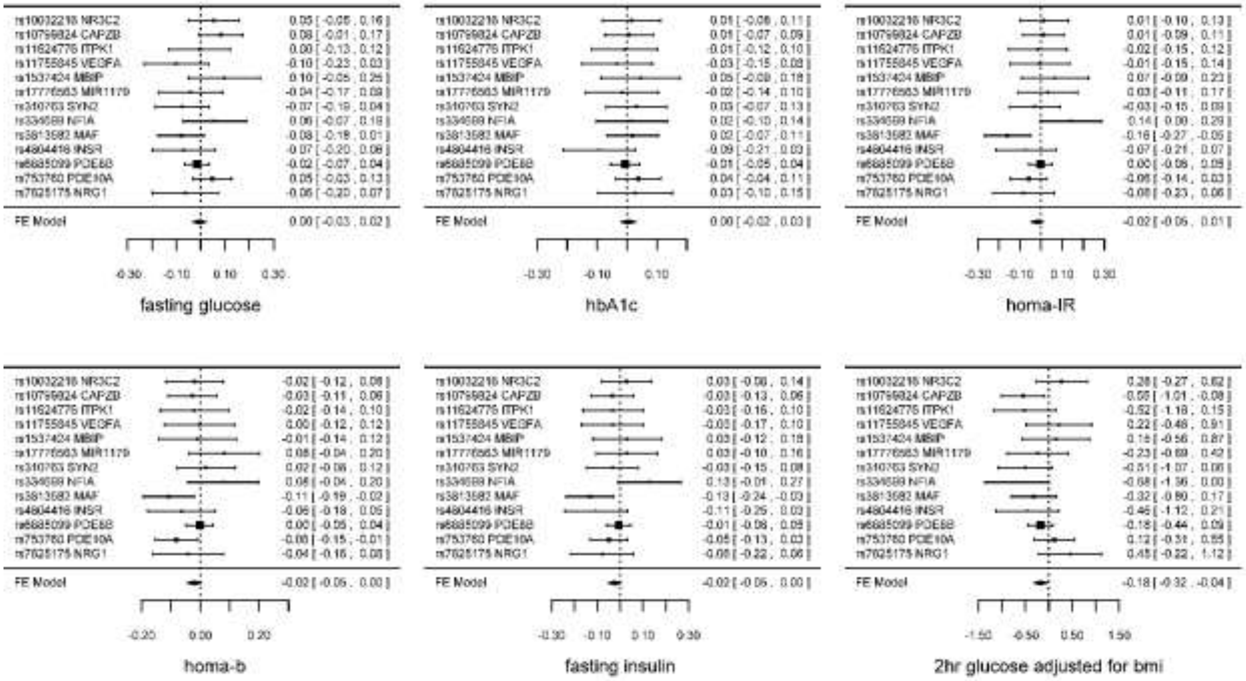

Supplemental Figure 2: SNP specific and total associations per standard deviation (SD) free thyroxine (FT4) with fasting glucose, hbA1c, log-transformed HOMA-IR, log transformed HOMA-b, log transformed fasting insulin and 2 hour glucose adjusted for body mass index (BMI), obtained from separate sample instrumental variable analysis in MAGIC.[3,13,14]

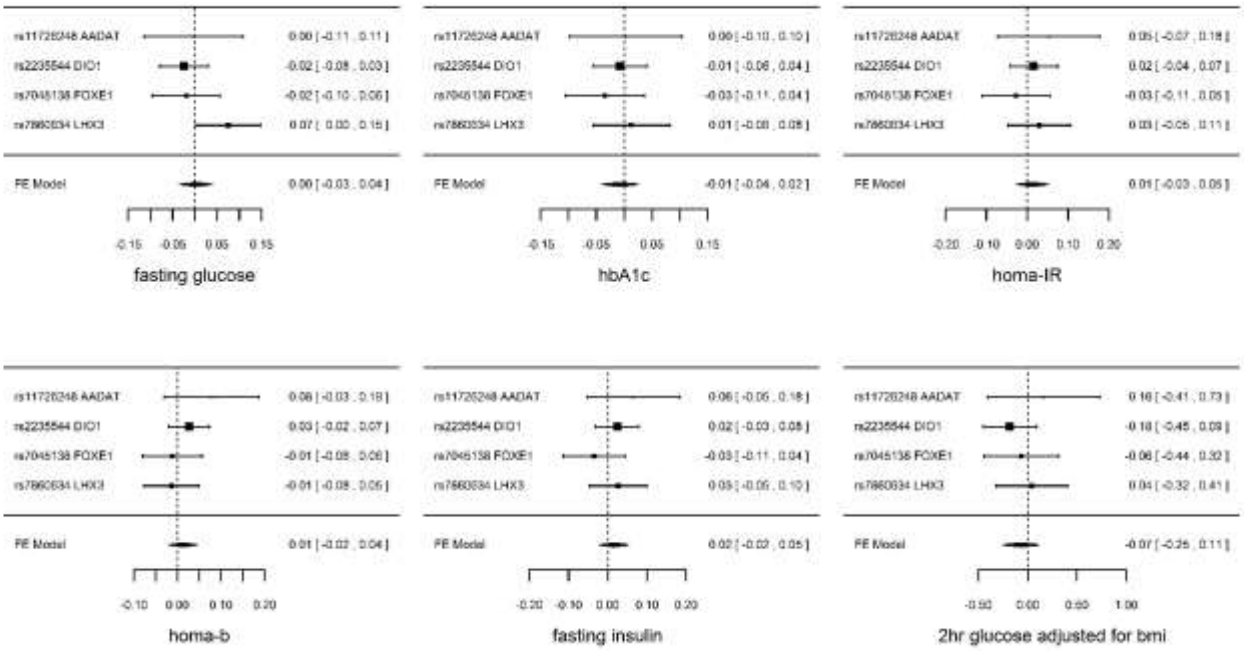

Supplemental Figure 3: SNP specific and total associations of thyroid peroxidase antibody (TPOAb) positivity with fasting glucose, hbA1c, log-transformed HOMA-IR, log transformed HOMA-b, log transformed fasting insulin and 2 hour glucose adjusted for body mass index (BMI), obtained from separate sample instrumental variable analysis in MAGIC.[3,13,14]

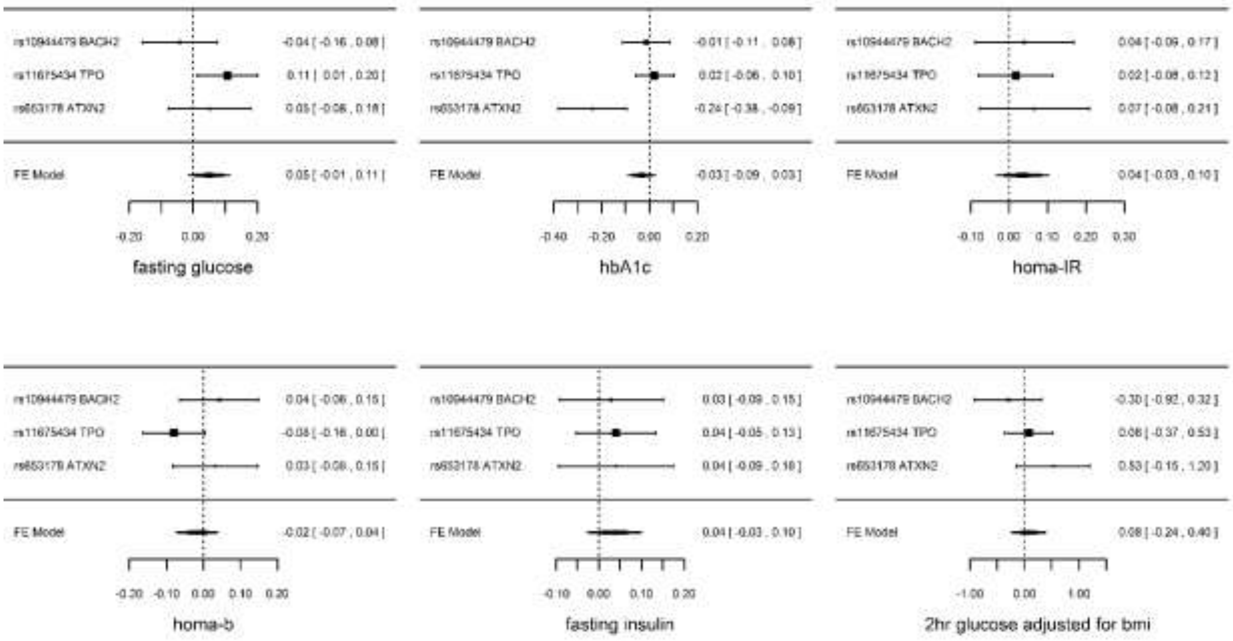

References

1 Nikpay, M. *et al.* A comprehensive 1,000 Genomes-based genome-wide association meta-analysis of coronary artery disease. *Nat. Genet.* **47**, 1121-1130 (2015).

2 Bradfield, J. P. *et al.* A genome-wide association meta-analysis identifies new childhood obesity loci. *Nat. Genet.* **44**, 526-531 (2012).

3 Dupuis, J. *et al.* New genetic loci implicated in fasting glucose homeostasis and their impact on type 2 diabetes risk. *Nat. Genet.* **42**, 105-116 (2010).

4 Randall, J. C. *et al.* Sex-stratified genome-wide association studies including 270,000 individuals show sexual dimorphism in genetic loci for anthropometric traits. *PLoS Genet.* **9**, e1003500 (2013).

5 Berndt, S. I. *et al.* Genome-wide meta-analysis identifies 11 new loci for anthropometric traits and provides insights into genetic architecture. *Nat. Genet.* **45**, 501-512 (2013).

6 Replication, D. I. G. *et al.* Genome-wide trans-ancestry meta-analysis provides insight into the genetic architecture of type 2 diabetes susceptibility. *Nat. Genet.* **46**, 234-244 (2014).

7 Kathiresan, S. *et al.* Common variants at 30 loci contribute to polygenic dyslipidemia. *Nat. Genet.* **41**, 56-65 (2009).

8 Global Lipids Genetics, C. *et al.* Discovery and refinement of loci associated with lipid levels. *Nat. Genet.* **45**, 1274-1283 (2013).

9 International Consortium for Blood Pressure Genome-Wide Association, S. *et al.* Genetic variants in novel pathways influence blood pressure and cardiovascular disease risk. *Nature* **478**, 103-109 (2011).

10 Rietveld, C. A. *et al.* GWAS of 126,559 individuals identifies genetic variants associated with educational attainment. *Science* **340**, 1467-1471 (2013).

11 Wain, L. V. *et al.* Genome-wide association study identifies six new loci influencing pulse pressure and mean arterial pressure. *Nat. Genet.* **43**, 1005-1011 (2011).

12 Morris, A. P. *et al.* Large-scale association analysis provides insights into the genetic architecture and pathophysiology of type 2 diabetes. *Nat. Genet.* **44**, 981-990 (2012).

13 Soranzo, N. *et al.* Common variants at 10 genomic loci influence hemoglobin A(1)(C) levels via glycemic and nonglycemic pathways. *Diabetes* **59**, 3229-3239 (2010).

14 Scott, R. A. *et al.* Large-scale association analyses identify new loci influencing glycemic traits and provide insight into the underlying biological pathways. *Nat. Genet.* **44**, 991-1005 (2012).

15 Locke, A. E. *et al.* Genetic studies of body mass index yield new insights for obesity biology. *Nature* **518**, 197-206 (2015).

16 Shungin, D. *et al.* New genetic loci link adipose and insulin biology to body fat distribution. *Nature* **518**, 187-196 (2015).
